# Ionizing radiation has negligible effects on the age, telomere length and corticosterone levels of Chornobyl tree frogs

**DOI:** 10.1098/rsbl.2024.0287

**Published:** 2024-11-06

**Authors:** Pablo Burraco, Caitlin Gabor, Amanda Bryant, Vanessa Gardette, Thierry Lengagne, Jean Marc Bonzom, Germán Orizaola

**Affiliations:** ^1^ Doñana Biological Station, Spanish National Research Council (EBD-CSIC), Sevilla 41092, Spain; ^2^ Department of Biology, Texas State University, San Marcos, TX 78666, USA; ^3^ Laboratoire d’Ecologie des Hydrosystèmes Naturels et Anthropisés (LEHNA), UMR 5023, CNRS, ENTPE, Université Claude Bernard Lyon 1, Villeurbanne F-69622, France; ^4^ Institut de Radioprotection et de Sûreté Nucléaire (IRSN), PSE-ENV/SERPEN/LECO, Cadarache, Saint Paul Lez Durance 13115, France; ^5^ Biodiversity Research Institute (IMIB), CSIC—University of Oviedo—Principality of Asturias, Mieres, Asturias 33600, Spain; ^6^ Zoology Unit, Department of Biology of Organisms and Systems, University of Oviedo, Oviedo, Asturias 33071, Spain

**Keywords:** ageing, amphibians, Chornobyl, contamination, lifespan, stress

## Abstract

The accident that occurred at the Chornobyl nuclear power plant (Ukraine, 1986) contaminated a large extension of territory after the deposition of radioactive material. It is still under debate whether the chronic exposure to the radiation levels currently present in the area has long-term effects on organisms, such as decreases in longevity. Here, we investigate whether current levels of radiation in Chornobyl negatively impact the age of the Eastern tree frog *Hyla orientalis*. We also explore whether radiation induces changes in an ageing marker, telomere length or the stress hormone corticosterone. We found no effect of total individual absorbed radiation (including both external and internal exposure) on frog age (*n* = 197 individuals sampled in 3 consecutive years). We also did not find any relationship between individual absorbed radiation and telomere length, nor between individual absorbed radiation and corticosterone levels. Our results suggest that radiation levels currently experienced by Chornobyl tree frogs may not be high enough to cause severe chronic damage to semi-aquatic vertebrates such as this species. This is the first study addressing age and stress hormones in Chornobyl wildlife, and thus future research will confirm if these results can be extended to other taxa.

## 1. Introduction

Ionizing radiation can directly damage DNA or interact with other molecules in the cell, causing indirect damage to DNA and even compromising organ function or individual survival [1]. Radiation can also lead to the upregulation of repair or buffering pathways such as repair mechanisms or antioxidants; however, these responses can be energetically costly and insufficient to fully avoid radiation effects, which could therefore result in lifespan reductions [2]. The accident at the Chornobyl nuclear power plant on 26 April 1986 led to the largest release of radioactive material to the environment ever recorded. While the consequences of this accident were severe in the short term for both humans and wildlife [3,4], it remains elusive whether current radiation levels have the potential to shape the ecology and evolution of wild populations in the contaminated areas. Recent research has reported a wide diversity of consequences of Chornobyl radiation on wild organisms, from severe impacts at the genetic and physiological level (e.g. [5–7]) to lack of effects and even the presence of abundant wildlife populations [8–11]. More than three decades have passed since the accident, radiation levels have dropped more than 90% overall, and short-lived radionuclides known to induce significant biological damage have completely disappeared from the area (e.g. ^131^I; [12]). There is a clear need for further studies aiming to address whether chronic exposure to current Chornobyl radiation (i.e. currently much lower than at the time of the accident and non-lethal in the short term) can lead to changes in animal life histories and health.

Organisms permanently exposed to ionizing radiation may experience negative effects on different fitness components such as survival and reproduction (e.g. [13]). However, no field-based study has addressed whether chronic exposure to radiation shapes age in Chornobyl wildlife so far. Shifts in individual age can have demographic implications including effects on population demographics [14,15] and can inform about the cumulative impact of coping with radiation over the course of life. This information can be complemented with markers of individual ageing or stress such as telomere length and corticosterone levels, respectively. Telomeres are the non-coding terminal sequences of the chromosomes that shorten as a consequence of environmental harshness, and thus, telomere shortening is often considered a marker of organismal ageing [16]. Anthropogenic disturbances are known to have a negative impact on the telomeres of some taxa [17,18], including ionizing radiation both on wildlife and humans ([19,20], but see [21]). However, this process seems to be context-dependent (reviewed in [18]). Likewise, corticosterone, the main glucocorticoid in amphibians, plays a key regulatory role in stress-induced responses [22–24]. Elevated chronic corticosterone levels can enhance metabolism, induce immunosuppression, and oxidative stress and, ultimately, have a negative impact on individual performance [25–28]. Investigating both telomere length and corticosterone levels can help understand the effects of radiation on animal ageing and health.

Here, we investigate whether current radiation levels in Chornobyl shape the age, telomere length and corticosterone levels of the Eastern tree frog *Hyla orientalis*. Our previous results on the effects of radiocontamination on *H. orientalis* in the Chornobyl region, conducted more than 30 years after the Chornobyl nuclear accident, showed no significant effects of radiocontamination on several physiological parameters. This includes unaltered blood biochemistry [10], markers of liver function [29] or the redox status of the frogs [30]. However, we detected an elevated mutation rate in the frogs living in the area compared with other European populations, with notably the presence of stop-gained mutations in the most contaminated areas and changes in the transcriptional profile in genes involved in energetic metabolism [31,32]. In this context, a chronic exposure to (relatively) low levels of ionizing radiation present in the Chornobyl region could have an impact on the lifespan of individuals. To explore this idea, we carried out an extensive sampling over 3 consecutive years in which we quantified individual age and absorbed dose rates in tree frogs inhabiting a gradient of radiation across the Chornobyl area. In a subset of individuals, we investigated individual ageing rate through estimates of telomere length and recorded levels of the stress hormone corticosterone. We expected that highly radiocontaminated frogs would be, overall, younger than those experiencing lower radiation levels. We also predicted shorter telomeres and higher corticosterone levels in frogs coping with high radiation levels.

## 2. Methods

### (a) Field sampling

The Eastern tree frog (*H. orientalis*) is a cryptic species of the European tree frog (*Hyla arborea*) group found in Asia Minor and southeastern Europe [33]. We captured reproductive adult males of this species in ponds located in the Chornobyl area (both within and outside the Chornobyl Exclusion Zone; electronic supplementary material, figure S1) in mid-May across 3 consecutive years (i.e. 2016–2018). We sampled 84 frogs in 2016, 70 in 2017 and 102 in 2018, for a total of 256 individuals sampled in 14 different localities across a marked gradient of ambient radiation within the Chornobyl area (electronic supplementary material, figure S1 and table S1). We estimated total individual absorbed dose rates for each frog integrating radionuclide activity concentrations in frogs (for ^137^Cs in muscle and ^90^Sr in bones), soil and water with dose coefficients (in μGy h^−1^ Bq^−1^ unit of mass^−1^, using a theoretical habitat use scenario during the breeding season on EDEN v3 IRSN software) and summing internal and external dose rates (see [34] for details). We also used ambient radiation levels to classify the localities included in this study in two categories: low (0.01–0.27 µSv h^−1^) and medium–high radiation (1.09–32.4 µSv h^−1^; [34]) to compare the results in our study system with previous ecological studies conducted in the area (e.g. [35,36]).

Frogs were captured during the night, transported to the laboratory located in Chornobyl town (Ukraine) and individually placed in buckets containing 5 cm of clean water until the next morning. Then, we recorded individual snout-to-vent length and width using a calliper, and we weighed each individual using a precision balance to the nearest 0.01 g. We euthanized frogs by pithing without decapitation (following AVMA guidelines [37]). From each frog, we collected the femur of the right hindlimb for age estimation and stored it in 70% ethanol until processing. In 2018, before euthanasia, we introduced a small ball of cotton in the mouth of frogs for approximately 15 s for saliva corticosterone quantification (*n* = 54 frogs). We also collected a portion of muscle from the right hindlimb for telomere length quantification (*n* = 62 frogs). All animals were collected, and experimental procedures were conducted, under the permit of the Ministry of Ecology and Natural Resources of Ukraine (no. 517, 21 April 2016).

### (b) Individual age

Skeletochronology allows estimating the age of animals with indeterminate growth living in temperate climates, by making use of the annual growth rings formed when osteogenesis is low or inactive. Such rings are defined by lines called lines of arrested growth (LAGs). Males of *H. orientalis* are mostly inactive during the cold months, which makes them good candidates for skeletochronology studies ([38], validated for hylid frogs by [39]).

We sampled femur bones that were decalcified in 1% nitric acid for 20 min and rinsed. Cross sections of the diaphyseal region of the bone were obtained using a freezing microtome (Microm Heidelberg HM330). We obtained several 18–20 μm sections and stained them with Ehrlich’s haematoxylin. We examined sections and quantified the number of LAGs on one section using a light microscope (Olympus CX40). Individual age was estimated by counting the number of lines following the first identifiable LAG (always by the same researcher, V.G.), which is preceded by a well-developed layer of bone [38]. The LAG of the first winter after metamorphosis is assumed to be very thin and is likely to be reabsorbed [38]; therefore, we added 1 year to the counted number of LAG.

### (c) Relative telomere length

We isolated genomic DNA using a commercial kit (DNeasy Blood & Tissue Kit; QIAGEN) and stored it at −80°C until assayed. We estimated relative telomere length through real-time quantitative polymerase chain reaction (qPCR). This method compares the amount of telomeric sequences relative to a control single-copy gene [40,41]. In our study, we amplified the ribosomal 18S gene as the qPCR control gene and the telomeric region using standard primers and qPCR conditions (see electronic supplementary material). We calculated qPCR plate efficiencies and ran melt curves demonstrating single peaks. We ran all samples in duplicate and calculated relative telomere length according to [42]. The intra-plate CV% was, respectively, 0.87% and 1.56% for 18S and telomere amplifications. Efficiency was, respectively, 98.11% and 94.92% for 18S and telomere amplifications. For each gene, samples were run in a single 384-well plate.

### (d) Corticosterone

We extracted and quantified saliva corticosterone following [43]. To quantify saliva volume, individual swabs were thawed, placed above a plastic filter in microcentrifuge tubes and centrifuged at 10 000 rpm for 10 min. We measured saliva volume from the bottom of each tube using a syringe (rinsed three times with EtOH and then three times with ddH_2_O between samples) and left it in the original microcentrifuge tube. Mean saliva volume was 3.27 ± 0.44 μl. We then washed each swab with 300 μl of EIA buffer into the original microcentrifuge tube to increase the yield of corticosterone from saliva swabs and centrifuged again at 10 000 rpm for 10 min. We added trichloroacetic acid to precipitate salivary proteins at a ratio of 20% of saliva volume [44]. We then vortexed samples for 10 s, incubated them at room temperature for 30 min, centrifuged them at 10 000 rpm for 10 min, and finally supernatants were transferred to a new microcentrifuge tube. We stored purified and concentrated samples at −20°C until assayed. We used a commercial EIA kit to quantify salivary corticosterone (item no. 501320; Cayman Chemical Company). Samples were assayed in duplicate read on a spectrophotometer plate reader at 405 nm (BioTek 800XS). Inter-plate variation was 8.82% for two plates. Methods to quantify corticosterone concentration in saliva have been validated in several amphibian species [43–45].

### (e) Statistical analyses

All analyses were conducted in R software (v. 4.2.1). We checked the appropriateness of our models with the help of the R package DHARMa (v. 0.4.6). We also tested if any spatial autocorrelation is present in our data (Matern covariance structure, through estimates of Moran’s I) with the help of the R package spaMM (v. 4.5.0). We obtained individual body condition estimates by getting the residuals of the linear relationship between body mass and length and also using the scaled mass index [46]. Body condition can correlate with absorbed dose rates in Chornobyl tree frogs [32]; hence, we tested whether this was the case for our dataset. Since we found that body condition estimates did not correlate with total dose rate (*p*-values > 0.09), we did not include this variable in further analyses. To study the relationship between individual absorbed dose rate and age, we conducted a linear mixed model that included locality nested within a year of sampling as the random factor. To investigate the effect of ambient radiation on age, we conducted a linear mixed model including age as the dependent variable, ambient radiation category as the independent category and locality nested within a year of sampling as the random factor. We tested for the relationships between telomere length, individual absorbed dose rate and age through a linear model including the three variables. Finally, we ran a linear model to test for an effect of individual absorbed dose rate on corticosterone levels, which also included individual age. We only included age estimates considered to have a high degree of reliability (197 out of 256 sampled individuals).

## 3. Results

**Figure 1.**
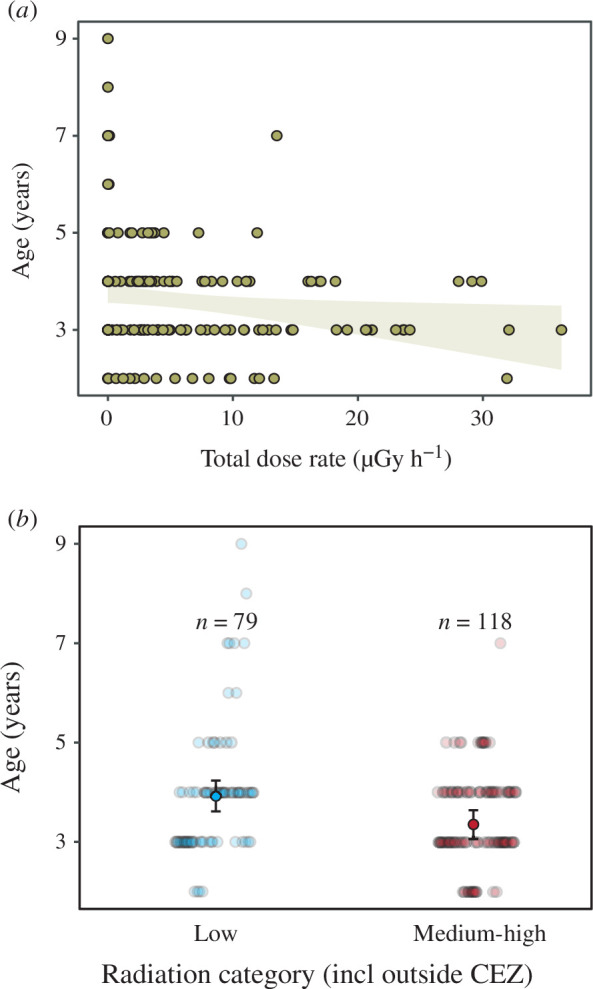
(*a*) Correlation between frog age and total individual dose rates in Eastern tree frog (*H. orientalis*) males sampled within the Chornobyl area (electronic supplementary material, figure S1). Since the correlation is not significant, the regression line is not drawn and grey lines indicate the 95% confidence interval. (*b*) Age difference between frogs inhabiting localities with low (0.01–0.27 µSv h^−1^) and medium–high radiation (1.09–32.4 µSv h^−1^).

**Figure 2.**
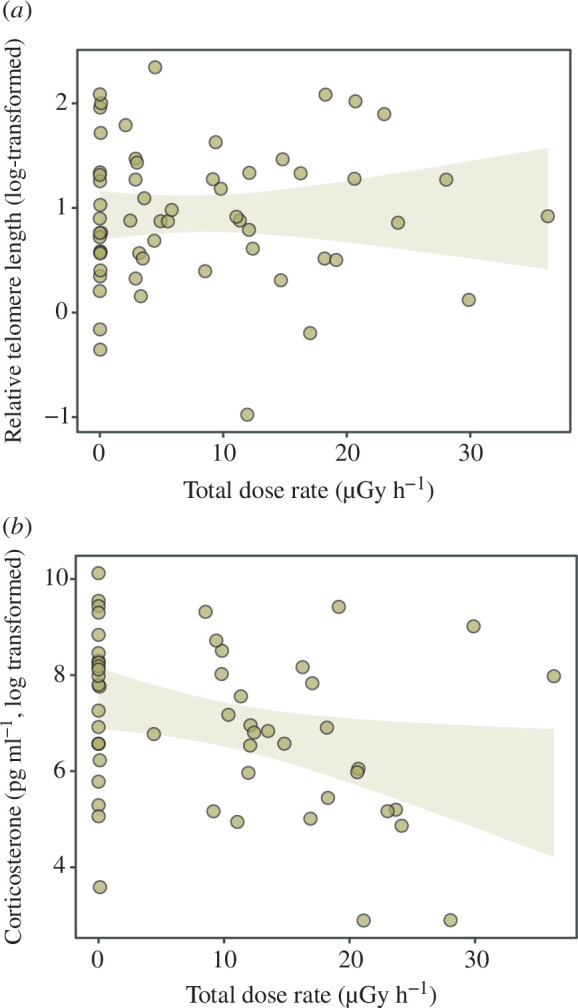
Correlation between frog age and (*a*) relative telomere length and (*b*) corticosterone levels in Eastern tree frog (*H. orientalis*) males sampled within the Chornobyl area (electronic supplementary material, figure S1). Since the correlations are not significant, regression lines are not drawn, and grey lines indicate the 95% confidence interval.

Frog age varied between 2 and 9 years (electronic supplementary material, table S1; Figure 1; see electronic supplementary material, figure S2 for variation in age proportion across localities), we found no significant correlation between total individual absorbed dose rate and frog age (*Χ*_1,177_ = 2.22, *p*‐value = 0.136, *R*^2^_m_ = 0.04; *R*^2^_c_ = 0.40; Figure 2*a*). Frogs inhabiting locations with medium–high ambient radiation levels were only marginally non-significantly younger (3.99 versus 3.44 years, i.e. 13.79% younger on average; Figure 2*b*) than those from populations with levels close to background (‘radiation category’ effect: *Χ*_1,196_ = 3.25, *p*‐value = 0.072, *R*^2^_m_ = 0.05; *R*^2^_c_ = 0.37; Figure 2*b*). Relative telomere length was not affected by individual absorbed dose rates (*Χ*_1,58_ = 0.01, *p*‐value = 0.923, *R*^2^_m_ = 0.01; *R*^2^_c_ = 0.19; Figure 1*a*), nor age (*Χ*_1,58_ = 0.01, *p*‐value = 0.99). Finally, corticosterone levels did not correlate with individual absorbed dose rates (Χ_1,51_ = 1.26, *p*‐value = 0.262, *R*^2^_m_ = 0.09; *R*^2^_c_ = 0.27; Figure 1*b*), and it was not affected by age (Χ_1,51_ = 0.21, *p*‐value = 0.647).

## 4. Discussion

Our study shows that radiation levels absorbed by male tree frogs in Chornobyl had no effect on the age of the frogs. When considering only ambient radiation, individuals from localities with high radiation levels were, overall, slightly younger than frogs from localities much less affected by radiation (although this tendency was non-significant). Radiation did not affect ageing rates in relation to the lack of variation in telomere length among individuals. Likewise, male frogs experiencing high dose rates showed unaltered corticosterone levels. This research represents the first approach for the study of the effects of chronic radiation exposure on the age and stress hormones in Chornobyl wildlife.

The magnitude of the impact caused by the chronic exposure to radiation in an organism is driven by the total dose absorbed by an organism over a period of time. In Chornobyl, radiation has decreased considerably since the moment of the accident [12], and nowadays, there is controversy about the effects of current radiation levels on wildlife (e.g. [47]). In our study system, Chornobyl tree frogs inhabiting radiocontaminated areas show normal physiological conditions ([10,29,30], but see [31,32], addressing effects at the genetic or transcriptomic level). In accordance with our previous physiological approaches in the same study system, we did not find a marked relationship between individual absorbed dose rates and frog age. Furthermore, our findings are based on estimates of the radiation absorbed by each individual frog, including both external and internal radiation (for more details, see [34]). Our study suggests that current radiation levels experienced by tree frogs in Chornobyl are not enough to markedly shorten their lifespan and agree with a previous study conducted on a similar species in radiocontaminated areas around Fukushima (Japan; [48]). Also, previous studies have shown that radiation doses currently experienced by tree frogs in Chornobyl are below the threshold considered harmful for amphibians [34], which may explain the little impact observed on the age of frogs.

When considering only environmental radiation levels, as done in many other studies in Chornobyl [35,36,49], and grouping localities with contrasting radiation levels (i.e. low and medium–high radiation), we found that frogs were slightly younger in highly contaminated localities than in those areas with radiation levels currently close to background. This result might suggest negative organismal impacts of early-life environmental conditions, parental effect or selective disappearance. However, the use of environmental radiation only is suboptimal, prevents the consideration of differences in individual exposure to radiation and extracting further conclusions. Age values recorded in our study are within the age range observed in the same species or in closely related species such as *H. arborea* in areas never exposed to radioactive fallout [39,50–52]. Hence, it seems that radiation has not caused a decrease in frog survival more than 30 years after the accident, but longitudinal approaches are still needed to fully disentangle it. A study conducted early after the accident showed a marked reduction in lifespan in experimental white mongrel rats from Chornobyl town and exposed to high radiation levels [53]. In humans, studies have shown, for example, evidence of increased risk of haematological malignancies and the development of cataracts or cardiovascular diseases caused by radiation [54,55], but the effect of chronic radiation exposure on the age structure of human populations has not been investigated yet. Long-term research should focus on increasing our understanding of possible age-dependent effects of chronic exposure to radiation on the fitness and health of different taxa [56].

Individual absorbed dose rates also do not seem to impact the ageing rate of Chornobyl tree frogs, based on its relation to telomere length. Since radiation does not seem to affect frog age, the lack of correlation between radiation and telomere length may further support that finding. The actions of different mechanisms protecting from telomere attrition, such as the enzyme telomerase or the antioxidant machinery, might be also behind the observed pattern. Telomerase expression is upregulated in some tissues in bank voles coping with Chornobyl radiation [57]. Likewise, Chornobyl tree frogs with high dose rates do not experience higher lipid peroxidation levels [30], which may indicate physiological compensatory responses to radiation similar to those observed in other amphibians coping with detrimental conditions earlier in their life [58]. We should also notice that we measured telomere length in the hindlimb muscle of frogs, but telomere dynamics can be tissue-specific in amphibians and other vertebrates [57,59,60]. Indeed, telomere dynamics differ across tissues in bank voles from the Chornobyl Exclusion Zone [57]. In agreement with our results, no damage on DNA, including telomeres, was recorded in wild boars and snakes exposed to radiation released after the Fukushima nuclear accident (Japan; [21]).

Finally, long-term exposure to environmental perturbations may lead to chronic stress, which can result in either up or downregulated corticosterone release, both known to cause detrimental effects on organisms [61–63]. In our study, radiation did not affect chronic corticosterone levels in male frogs. Here, frogs were maintained in the lab overnight before taking saliva samples for measuring the hormone, which may have induced corticosterone responses across sampled frogs. Capture and transportation to the lab is stressful to amphibians, and thus, frogs from all sites may have been similarly stressed [64–66]. The study of stress hormones has been overlooked in Chornobyl research (already highlighted by [36]), and thus, future work should determine if our findings can be extended to other taxa.

## 5. Conclusions

Our study finds no effect of ionizing radiation levels currently experienced by male tree frogs inhabiting Chornobyl on their age, nor on an ageing marker, telomere length, or the stress hormone, corticosterone. These results seem to be in line with our previous work on different phenotypic traits in the same study system and suggest that current Chornobyl radiation levels are not high enough to cause chronic damage to tree frogs.

## Data Availability

Open research statement data are available at [67]. Supplementary material available online [68].
